# Comparing [18F]FET PET and [18F]FDOPA PET for glioma recurrence diagnosis: a systematic review and meta-analysis

**DOI:** 10.3389/fonc.2023.1346951

**Published:** 2024-01-10

**Authors:** Pengbo Yu, Yinan Wang, Fengbo Su, Yan Chen

**Affiliations:** Department of Neurosurgery, The Second Hospital of Jilin University, Changchun, China

**Keywords:** glioma, [18F]FET PET, [18F]FDOPA PET, recurrence, meta-analysis

## Abstract

**Purpose:**

The purpose of our meta-analysis and systematic review was to evaluate and compare the diagnostic effectiveness of [18F]FET PET and [18F]FDOPA PET in detecting glioma recurrence.

**Methods:**

Sensitivities and specificities were assessed using the DerSimonian and Laird methodology, and subsequently transformed using the Freeman-Tukey double inverse sine transformation. Confidence intervals were computed employing the Jackson method, while heterogeneity within and between groups was evaluated through the Cochrane Q and I² statistics. If substantial heterogeneity among the studies was observed (P < 0.10 or I² > 50%), we conducted meta-regression and sensitivity analyses. Publication bias was assessed through the test of a funnel plot and the application of Egger’s test. For all statistical tests, except for assessing heterogeneity (P < 0.10), statistical significance was determined when the two-tailed P value fell below 0.05.

**Results:**

Initially, 579 publications were identified, and ultimately, 22 studies, involving 1514 patients(1226 patients for [18F]FET PET and 288 patients for [18F]FDOPA PET), were included in the analysis. The sensitivity and specificity of [18F]FET PET were 0.84 (95% CI, 0.75-0.90) and 0.86 (95% CI, 0.80-0.91), respectively, while for [18F]FDOPA PET, the values were 0.95 (95% CI, 0.86-1.00) for sensitivity and 0.90 (95% CI, 0.77-0.98) for specificity. A statistically significant difference in sensitivity existed between these two radiotracers (P=0.04), while no significant difference was observed in specificity (P=0.58).

**Conclusion:**

It seems that [18F]FDOPA PET demonstrates superior sensitivity and similar specificity to [18F] FET PET. Nevertheless, it’s crucial to emphasize that [18F]FDOPA PET results were obtained from studies with limited sample sizes. Further larger prospective studies, especially head-to-head comparisons, are needed in this issue.

**Systematic Review Registration:**

identifier CRD42023463476

## 1 Introduction

Glioma, a primary tumor of the central nervous system, represents a formidable challenge in the realm of oncology due to its infiltrative nature and variable biological behavior (1, 2).Nevertheless, a few months into treatment, numerous patients experience pseudoprogression or radiation necrosis, conditions frequently indistinguishable from tumor recurrence (3). Given the potential aggressiveness of glioma recurrence, early detection is paramount in facilitating interventions that can potentially extend patient survival and improve their quality of life (4).

Historically, conventional imaging modalities such as computed tomography (CT) and magnetic resonance imaging (MRI) have played a pivotal role in glioma diagnosis and monitoring (5).While these methods have provided essential insights into tumor structure and volume, they have shown limitations in distinguishing between active tumor tissue and post-treatment changes, often leading to equivocal results (6). CT scans utilize X-rays to create detailed cross-sectional images of the brain, allowing clinicians to visualize the tumor’s location, size, and its impact on surrounding structures. However, CT scans are limited in their capacity to differentiate different types of brain tissue with precision. This lack of specificity can lead to difficulties in distinguishing active tumor tissue from non-cancerous changes, such as post-treatment radiation effects or edema, which can yield false-positive results. MRI, a non-invasive imaging technique, offers superior soft tissue contrast and is especially valuable in delineating tumor boundaries and identifying associated brain edema (4). However, similar to CT, MRI also faces challenges when it comes to distinguishing between recurrent tumor and radiation-induced changes (7). Glioma recurrence can present with subtle changes that may overlap with post-treatment effects, causing diagnostic ambiguity (2, 8). These limitations have spurred the exploration of advanced imaging techniques that can offer improved specificity and sensitivity in detecting glioma recurrence (9, 10).

A significant development in this pursuit is the application of positron emission tomography (PET) imaging using radiolabeled amino acids like [18F]FET (O-(2-[18F]fluoroethyl)-L-tyrosine) and [18F]FDOPA (6-[18F]fluoro-L-DOPA). These radiotracers have shown promise in glioma recurrence diagnosis by capitalizing on the increased metabolic activity of tumor cells. [18F]FET is an amino acid analog that is actively transported into tumor cells, reflecting increased amino acid metabolism associated with malignancy (11, 12), known for its minimal uptake in normal brain tissue and rapid clearance from non-tumor cells, displays a distribution pattern predominantly focused within the tumor, enhancing the contrast between malignant and healthy tissues (13). Conversely, [18F]FDOPA PET relies on the radiotracer 6-[18F]fluoro-L-DOPA, which is a precursor of dopamine and is actively transported into cells (14). Like [18F]FET PET, [18F]FDOPA PET can detect regions of heightened metabolic activity, but it does so by targeting amino acid metabolism differently. [18F]FDOPA, on the other hand, shows a somewhat different biodistribution, characterized by a higher basal level of uptake in normal brain tissue but still demonstrates a significant increase in uptake in tumor cells (15). This distinction in biodistribution between [18F]FET and [18F]FDOPA is pivotal in their application for glioma recurrence detection and forms a basis for ongoing comparative studies. Some studies suggest that [18F]FET PET may offer superior diagnostic accuracy due to its specificity for amino acid transport, while others argue that [18F]FDOPA PET’s ability to probe different aspects of amino acid metabolism makes it a preferable choice (12, 14).

In light of the ongoing debate surrounding the diagnostic accuracy of [18F]FET PET and [18F]FDOPA PET in glioma recurrence, this systematic review and meta-analysis seek to provide a rigorous and evidence-based comparison of these imaging techniques. Our primary objective is to assess the diagnostic performance of [18F]FET PET and [18F]FDOPA PET in detecting glioma recurrence, including their sensitivity and specificity.

## 2 Materials and methods

Our review has been registered with PROSPERO, the international prospective register of systematic reviews, under the identifier CRD42023463476.

### 2.1 Search strategy

A comprehensive search was conducted of the PubMed and Embase databases for all available literatures through September 10, 2023 based on the following combination of terms:(1)Positron-Emission Tomography OR PET OR Positron-Emission Tomography; (2)Regeneration OR Recurrence OR pseudoprogression OR recurrent OR relapse OR Recrudescence OR radionecrosis;(3) Glioma OR Glioma OR Glial Cell Tumor OR Mixed Glioma OR Malignant Glioma;(4) fluoroethyltyrosine OR FET OR fluorodopa F-18 OR FDOPA OR fluorodopa OR 18F-dopa. Studies that were potentially related were also enclosed from the reference lists.

### 2.2 Inclusion and exclusion criteria

Only studies that met all of the following condition were included: (1) Articles evaluating the diagnostic efficiency of [18F]FET PET or [18F]FDOPA PET in detecting glioma recurrence; (2) Patients under suspicion of recurrent glioma, without any limitations related to age, gender, race, or geographical origin; (3) A prerequisite for inclusion is a minimum of 10 patients or lesions.; (3) The reference standard included histopathological confirmation or imaging follow-up, a requirement that should be explicitly stated in the article; (4) True positive (TP), false positive (FP), true negative (TN), false negative (FN) data could be extracted. The exclusion condition were: (1) Irrelevant topic; (2) Duplicated articles; (3) Cell or animal experiments; (4) Non-English articles; (5) Abstract, editorial comments, letters, case reports, review and meta-analyses. After reviewing the titles and abstracts of the articles based on the incorporation and exclusion criteria, we evaluated the full-text variants of the selected articles to confirm their adherence to the inclusion criteria. Any disagreements between scholars were solved by consensus.

### 2.3 Quality assessment and data extraction

Using the Quality Assessment of Diagnostic Performance Studies (QUADAS-2) methodology (16), two independent researchers assessed the quality of the included studies. They evaluated each study’s risk of bias and applicability, rating them as either high, low, or unclear in these aspects. In case of any disputes, a third reviewer was consulted for resolution. The analysis was conducted using RevMan (version 5.4).

Data extraction for all incorporated papers was carried out separately by two researchers(**Table 1**). The data that were extracted included: (1) The author, year of publication; (2) Study characteristics including country, study design, analysis, duration, reference standard; (3) Patient characteristics including variety of patients, mean/median age; (4) Technical characteristics including types of tracers, parameter, TP, FP, FN, TN. Data were manually accessed from the literature, tables, and figures when not clearly stated. If the article lacked sufficient information, we will contact the corresponding authors by email and request further data or interpretation. Any disagreements between the two researchers were consequently resolved by consensus.

**Table 1:** Characteristics of the included studies and patients.

Author	Year	Country	Type of tracers	Study duration	Study design	Analysis	Reference standard	Parameter	No. of patients	Mean/Median age	TP	FP	FN	TN
Vidmar et al.	2022	Slovenia	[18F]FET PET	2019-2021	Retro	LB	Pathology and follow-up imaging	TBRmax	47	Median(range):44(17-72)	9	7	2	24
Muller et al.	2022	Germany	[18F]FET PET	NA	Retro	LB	Pathology and follow-up imaging	TBRmean + TBRmax	151	Median(range):52(20-78)	8	9	4	37
Puranik et al.	2021	India	[18F]FET PET	2017-2019	Retro	PB	Pathology and follow-up imaging	T/Wm	72	NA	35	6	4	27
Paprottka et al.	2021	Germany	[18F]FET PET	2017-2020	Retro	LB	Pathology and follow-up imaging	TBRmean	66	Mean+SD:54.91 ± 12.2	14	11	3	46
Werner et al.	2021	Germany	[18F]FET PET	2018-2020	Retro	PB	Pathology and follow-up imaging	TBRmean	23	Mean+SD:58 ± 9	9	1	2	11
Steidl et al.	2021	Germany	[18F]FET PET	2016-2019	Retro	PB	Pathology and follow-up imaging	Slope	104	Median(range):52(20-78)	13	13	8	70
Lohmann et al.	2020	Germany	[18F]FET PET	NA	Pro	PB	Pathology and follow-up imaging	TBRmax	34	Mean ± SD:57 ± 12	13	6	3	12
Kebir et al.	2020	Germany	[18F]FET PET	NA	Retro	PB	follow-up imaging	TBRmean	44	Median(range):55(34-79)	5	0	9	30
Maurer et al.	2020	Germany	[18F]FET PET	2016-2019	Retro	PB	Pathology and follow-up imaging	TBRmax	127	Mean+SD:50 ± 12	23	28	10	66
Bashir et al.	2019	Denmark	[18F]FET PET	2011-2019	Pro	LB	Pathology and follow-up imaging	TBRmax	146	Median(range):59.5 (21–80)	150	1	2	15
Kertels et al.	2018	Germany	[18F]FET PET	2010-2016	Retro	PB	Pathology and follow-up imaging	TBR80%	36	Mean+SD:54 ± 14	23	1	5	7
Pyka et al.	2018	Germany	[18F]FET PET	2015-2017	Retro	LB	Pathology and follow-up imaging	FET 30-40	47	Mean+SD:53 ± 11	40	2	10	11
Galldiks et al.	2015	Germany	[18F]FET PET	2006-2013	Retro	LB	Pathology	TBRmean/TTP	124	Mean+SD:52 ± 14	113	0	8	11
Dunkl et al.	2015	Germany	[18F]FET PET	2006-2012	Retro	LB	Pathology	18F-FET kinetic pattern	18	Median(range):13 (1–18)	10	1	3	10
Herrmann et al.	2013	Germany	[18F]FET PET	NA	Retro	PB	Pathology and follow-up imaging	visual scale	110	Mean+SD:51.7 ± 12.1	69	8	12	21
Jeong et al.	2010	Korea	[18F]FET PET	2003-2009	Retro	PB	Pathology and follow-up imaging	SUVmax	32	Mean+SD:47.3 ± 10	20	0	3	9
Rachinger et al.	2005	Germany	[18F]FET PET	2001-2003	Retro	PB	Pathology and follow-up imaging	SUVmax	45	Mean+SD:45 ± 12	31	1	0	13
Rozenblum et al.	2022	France	[18F]FDOPA PET	2015-2020	Retro	PB	Pathology and follow-up imaging	TBRmean	106	Median:54	58	10	13	25
Pellerin et al.	2021	Germany	[18F]FDOPA PET	2015-2018	Pro	PB	Pathology and follow-up imaging	T-map and isocontour map	58	Mean+SD:53.1 ± 14.3	22	2	2	32
Li et al.	2021	China	[18F]FDOPA PET	2016-2019	Pro	PB	Pathology and follow-up imaging	L/G	43	Mean ± SD:38.5 ± 6.37	34	1	0	8
Zaragori et al.	2020	France	[18F]FDOPA PET	2012-2017	Retro	PB	follow-up imaging	TBRmax	51	Median(range):51(21-75)	16	1	1	33
Karunanithi et al.	2014	India	[18F]FDOPA PET	2009-2010	Pro	PB	Pathology and follow-up imaging	T/C	30	NA	22	1	0	7

PB, patient-based; LB, lesion-based; Pro, prospective; Retro, retrospective; NA, not available.

### 2.4 Data synthesis and statistical analysis

The sensitivities and specificities were evaluated using the DerSimonian and Laird method and transformed with the Freeman-Tukey double inverse sine transformation. The confidence intervals were calculated using the Jackson method. The Cochrane Q and I² statistics were used to assess the heterogeneity within and between groups. If the heterogeneity between the studies differed significantly (P < 0.10 or I² > 50%), meta-regression analysis and sensitivity analysis were performed by reassessing the sensitivities or specificities following the omission of articles one by one. This was done to evaluate the robustness of the overall sensitivities or specificities and to identify single studies that may contribute to heterogeneity.

Publication bias was assessed through a funnel plot and Egger’s test. Except for heterogeneity (P < 0.10), a two-tailed p-value below 0.05 was considered statistically significant for all statistical tests. Statistical analyses were performed using the R software for statistical computing and graphics version 4.3.1.

## 3 Results

### 3.1 Literature search and study selection

**Figure 1:**
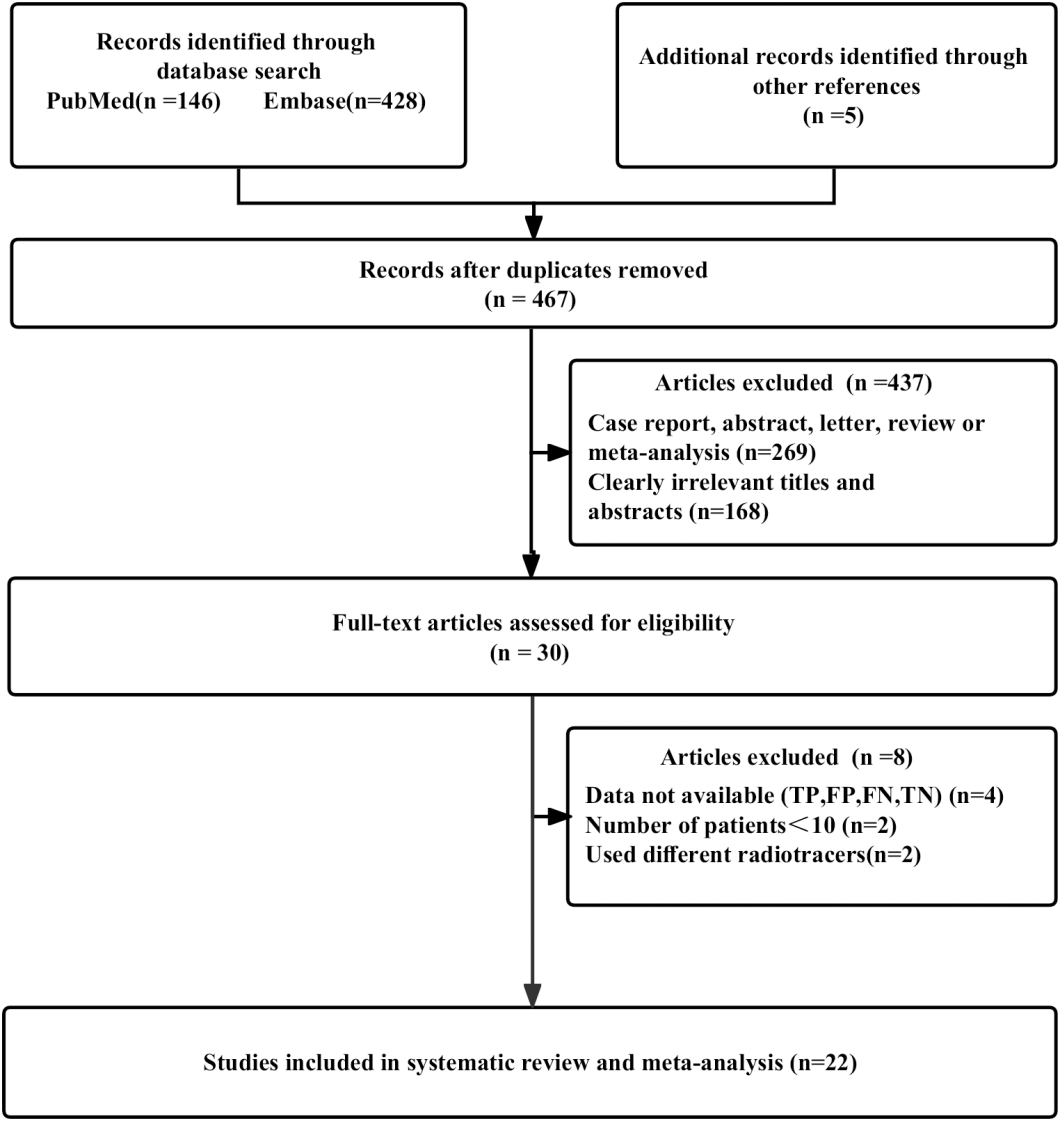
The PRISMA flow chart of investigation selection procedure.

The initial search yielded a total of 579 publications. After eliminating 112 duplicated studies, we identified 467 unique studies. Upon reviewing the titles and abstracts, 437 studies were excluded. Among the remaining results, 4 lacked available data, 2 had fewer than 10 patients, and 2 utilized different radiotracers. Finally, 22 studies assessing the diagnostic accuracy of glioma recurrence diagnosis, involving 1514 patients, were included in the analysis. This encompassed 17 articles specifically focusing on [18F]FET PET (11, 17–32) and an additional 5 articles centered on [18F]FDOPA PET (33–37). The PRISMA flow diagram of the study selection process was shown in **Figure 1**.

### 3.2 Study description and quality assessment study description and quality assessment

**Figure 2:**
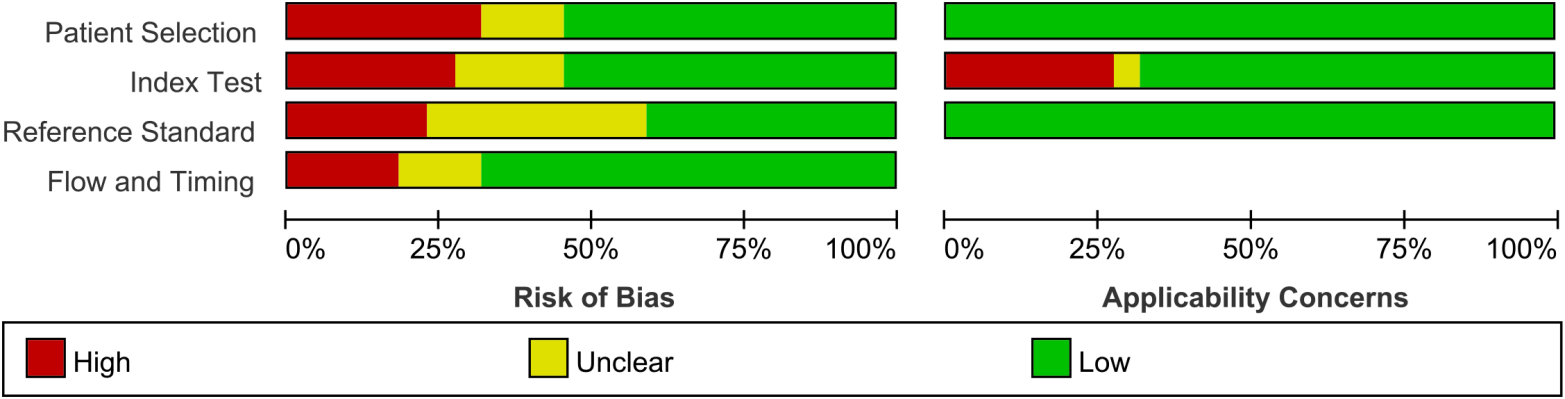
Risk of bias items presented as percentages across all articles using the QUADAS-2 tool.

**Table 1** presents the study characteristics and technical details derived from the 22 selected studies, encompassing a total of 1514 patients. Additionally, we conducted an assessment of the study quality, utilizing the Quality Assessment of Diagnostic Accuracy Studies (QUADAS-2) tool (16). The quality evaluation graph elucidated that the primary area of high-risk bias concerns was centered around patient selection (**Figure 2**), primarily due to the fact that many of the studies did not involve consecutive patient recruitment. In general, the risk of bias in the articles was deemed acceptable.

### 3.3 Diagnostic performance of [18F]FET PET and [18F]FDOPA PET for glioma recurrence

**Figure 3:**
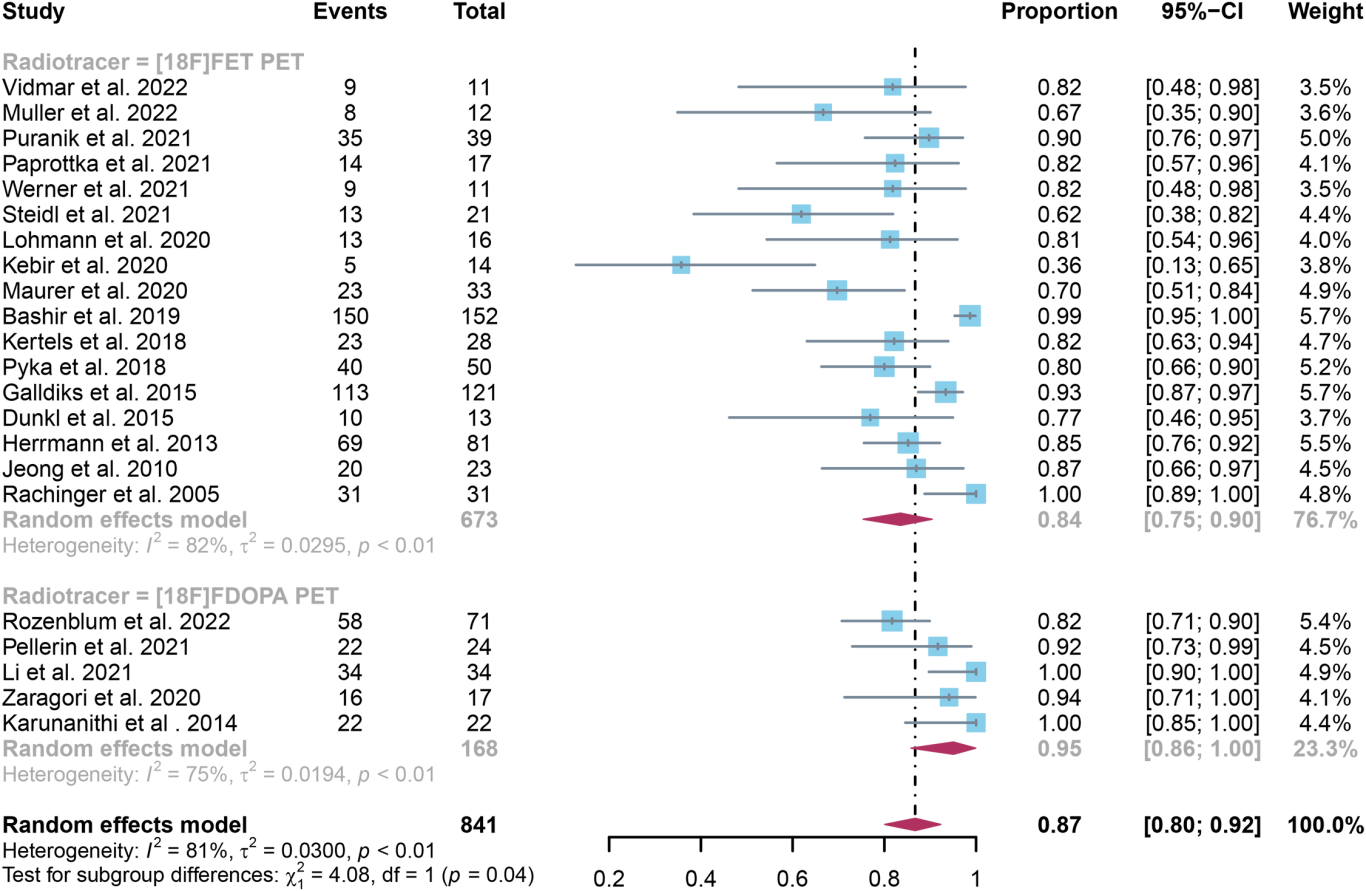
Forest plot comparing sensitivity of [18F]FET PET and [18F]FDOPA PET in glioma recurrence.

**Figure 4:**
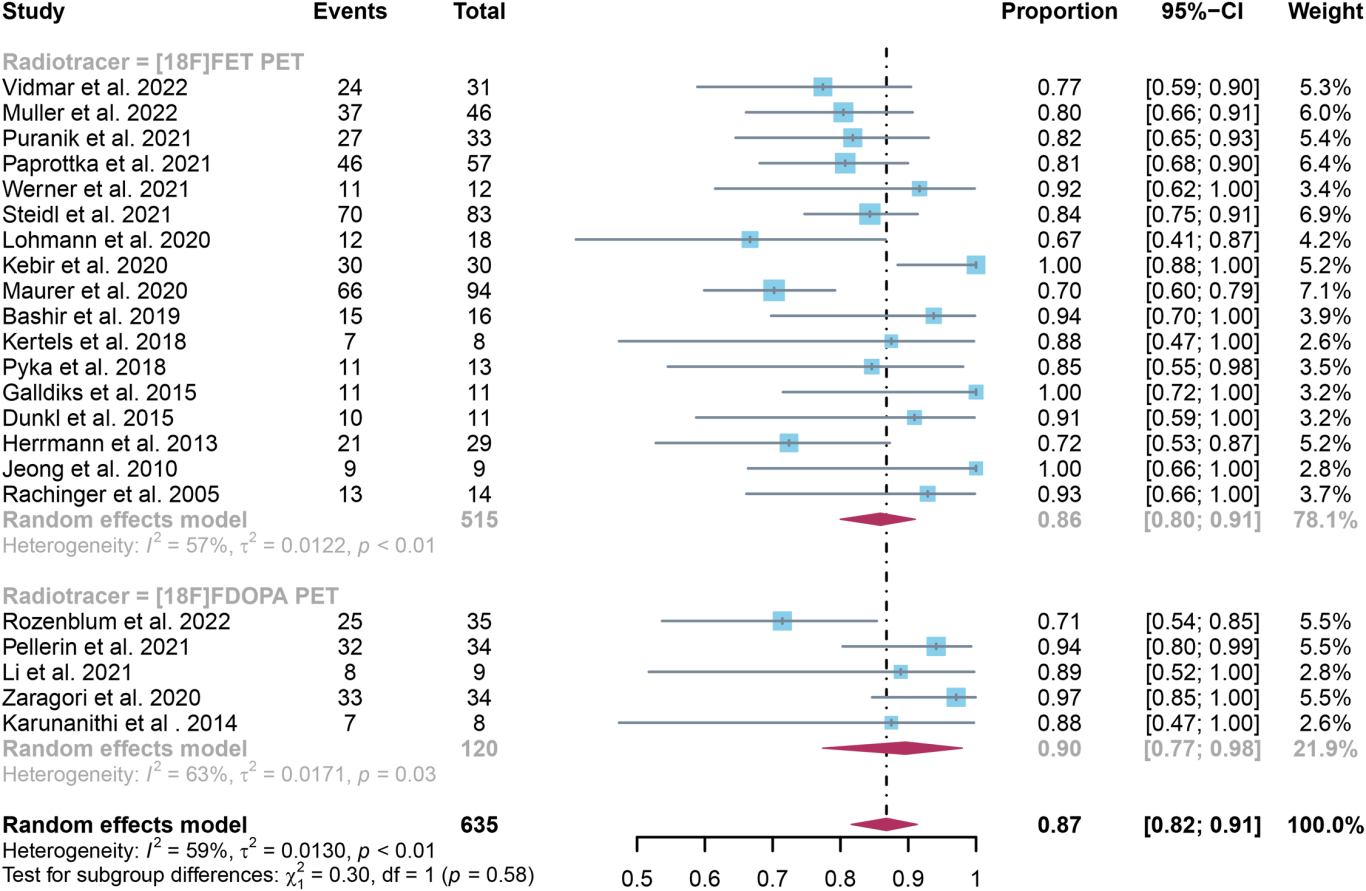
Forest plot comparing specificity of [18F]FET PET and [18F]FDOPA PET in glioma recurrence.

The pooled sensitivity for glioma recurrence was 0.84 (95% CI, 0.75-0.90) for [18F]FET PET and 0.95 (95% CI, 0.86-1.00) for [18F]FDOPA PET (**Figure 3**). Likewise, the pooled specificity for [18F]FET PET was 0.86 (95% CI, 0.80-0.91), while for [18F]FDOPA PET, it was 0.90 (95% CI, 0.77-0.98)(**Figure 4**). A statistically significant difference in sensitivity existed between these two radiotracers (P=0.04), while no significant difference was observed in specificity (P=0.58).

**Figure 5:**
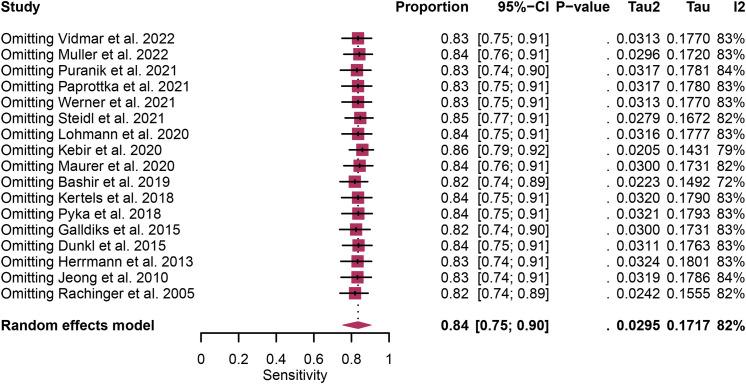
Sensitivity analysis evaluating heterogeneity in [18F]FET PET sensitivity for glioma recurrence diagnosis.

**Figure 6:**
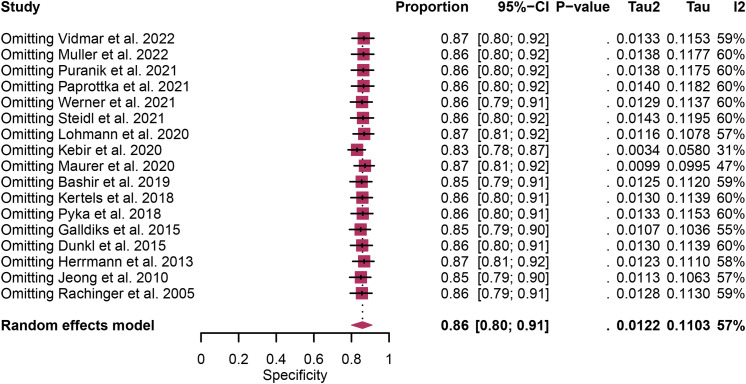
Sensitivity analysis evaluating heterogeneity in [18F]FET PET specificity for glioma recurrence diagnosis.

**Figure 7:**
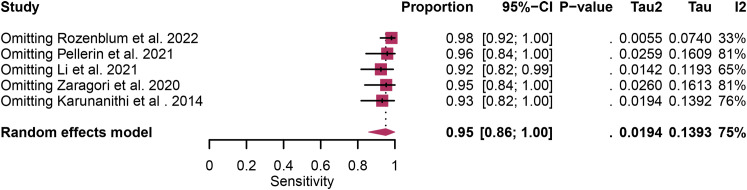
Sensitivity analysis evaluating heterogeneity in [18F]FDOPA PET sensitivity for glioma recurrence diagnosis.

**Figure 8:**
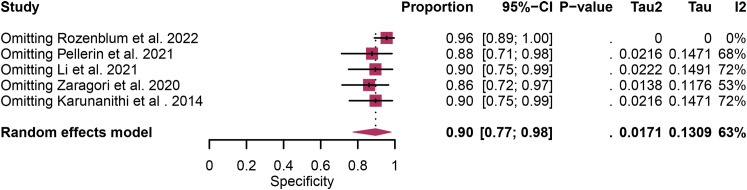
Sensitivity analysis evaluating heterogeneity in [18F]FDOPA PET specificity for glioma recurrence diagnosis.

Regarding the sensitivity of [18F]FET PET and [18F]FDOPA PET for glioma recurrence, the I^2^ was 82%, 75%, respectively. In terms of the specificity of [18F]FET PET and [18F]FDOPA PET, the I^2^ were 57% and 63%. For [18F] FET PET, we did not find the reason for its sensitivity heterogeneity through sensitivity analysis and meta-regression analysis (**Figure 5**) (**Table 2**). This may be related to significant differences in the study duration of different studies and many of the studies did not involve consecutive patient recruitment. The meta-regression analysis showed that the reference standard (P=0.01 for specificity) may account for the heterogeneity (**Table 2**). Sensitivity analysis, excluding data from Kebir et al. (25) and Maurer et al. (24),resulted in a combined specificity of 0.83 (95% CI: 0.78-0.87) and 0.83 (95% CI: 0.81-0.92) with low heterogeneity (I^2 = ^31% and I^2 = ^47%), respectively (**Figure 6**). For [18F]FDOPA PET, sensitivity analysis by excluding data from Rozenblum et al. (33) reported a combined specificity of 0.96(95% CI: 0.89-1.00), with an acceptable heterogeneity (I^2 = ^0%) (**Figure 7**). Sensitivity analysis, after the exclusion of data from Rozenblum et al. (33), yielded a combined sensitivity of 0.98 (95% CI: 0.92-1.00) with minimal heterogeneity (I^2 = ^33%) (**Figure 8**).

**Table 2:** Meta-regression analysis for [18F]FET PET in glioma recurrence.

Covariate	Studies, n	Sensitivity (95%CI)	*P*-value	Specificity (95%CI)	*P*-value
Number of patients included			0.78		0.43
>100	11	0.83(0.73-0.91)		0.88(0.80-0.94)	
≤100	6	0.84(0.68-0.95)		0.83(0.73-0.90)	
Race			0.58		0.59
White	15	0.83(0.73-0.91)		0.85(0.79-0.91)	
Yellow	2	0.89(0.79-0.96)		0.91(0.68-1.00)	
Study design			0.13		0.61
Retrospective	15	0.82(0.73-0.89)		0.86(0.80-0.92)	
Prospective	2	0.94(0.68-1.00)		0.82(0.50-1.00)	
Analysis			0.43		0.89
Patient-based	7	0.87(0.76-0.96)		0.85(0.79-0.90)	
Lesion-based	10	0.81(0.69-0.91)		0.86(0.77-0.94)	
Reference standard			0.13		0.01
Pathology and follow-up imaging	14	0.85(0.77-0.92)		0.82(0.77-0.86)	
Pathology	2	0.89(0.68-0.10)		0.89(0.68-1.00)	
Follow-up imaging	1	0.84(0.75-0.90)		1.00(0.86-1.00)	

### 3.4 Publication bias

The funnel plot asymmetry test revealed a significant publication bias regarding the sensitivity of [18F]FET PET, as indicated by Egger’s test (P=0.01), no significant publication bias was found in relation to the specificity of [18F]FET PET (P=0.06). No notable publication bias was observed in sensitivity and specificity for [18F]FDOPA PET (P=0.25, 0.86).

## 4 Discussion

The detection of recurrent signs during post-treatment follow-up for glioma patients portends an unfavorable prognosis. Several studies indicate that patients experiencing their first recurrence have a median survival time of only 9 to 10 months (38). The central question that has spurred extensive debate within the neuro-oncology community revolves around the optimal choice between [18F]FET PET and [18F]FDOPA PET for the diagnosis of glioma recurrence (14, 39–41).

It seems that [18F]FDOPA PET demonstrates superior sensitivity and similar specificity to [18F] FET PET. [18F]FET PET exhibits a pooled sensitivity of 0.84 (95% CI, 0.75-0.90) and specificity of 0.86 (95% CI, 0.80-0.91), while [18F]FDOPA PET demonstrates a pooled sensitivity of 0.95 (95% CI, 0.86-1.00) and specificity of 0.90 (95%CI, 0.77-0.98).These results underscore that both radiotracers are valuable tools in clinical practice (14). It seems that [18F]FDOPA PET demonstrates superior sensitivity in detecting glioma recurrence when contrasted with [18F]FET PET. This may be due to their slightly different mechanisms of action. [18F]FET, an amino acid analog, is transported into tumor cells via amino acid transporters. It capitalizes on the increased amino acid metabolism observed in malignant tissue (42). Conversely, [18F]FDOPA, a precursor of dopamine, is actively transported into cells and reflects increased amino acid metabolism as well. Its advantage lies in targeting different aspects of amino acid metabolism, which may contribute to its diagnostic sensitivity in distinguishing between recurrent tumor and treatment-related changes (43).

Despite numerous published studies, the selection of the ideal radiotracer for discriminating between authentic glioma recurrence and spurious progression remains undetermined. Previously, two meta-analyses regarding [18F]FET PET or [18F]FDOPA PET for glioma recurrence have been conducted and published. According to a meta-analysis by Yu et al. (14), the findings suggest that [18F]FDOPA PET exhibited superior diagnostic performance in patients with glioma recurrence. In summary, within the glioma subgroup, [18F]FDOPA PET demonstrated superior ability across all outcomes compared to [18F]FET PET: sensitivity (0.94 vs. 0.78) and specificity (0.89 vs. 0.75). However, in this article, all data pertaining to the diagnosis of glioma recurrence using [18F]FDOPA PET were sourced from a compilation of three studies (comprising 10 studies) conducted by the same research institution and authored by Karunanithi et al. (37, 43, 44) This circumstance could potentially undermine the reliability of the research data, consequently impacting the outcomes of subgroup analysis. In 2023, Tian et al (12). conducted a systematic review and meta-analysis of the diagnostic performance of different PET imaging agents for glioma recurrence. They included 15 articles that met the inclusion criteria, and ultimately showed that [18F]FET had the highest SUCRA values (diagnostic performance) in sensitivity, specificity, positive predictive value, and accuracy, followed by 18F-FDOPA. Indicating that [18F]FET is one of the most popular imaging agents for glioma recurrence. However due to the limitations of network meta-analysis, articles that only evaluate individual [18F]FET PET or [18F]FDOPA PET were not included, resulting in many available articles being excluded, further affecting the credibility of their articles. While prior meta-analyses have explored this topic to varying degrees, several factors differentiate our study and make it a valuable addition to the existing body of literature. One of the critical strengths of our analysis is the incorporation of the most recent and up-to-date studies (9, 17–22, 33–35). This inclusion ensures that our findings are aligned with the latest research developments, providing the most relevant insights for clinical practice.

Heterogeneity is an inherent challenge in meta-analyses, and it was indeed observed in our study, there was high heterogeneity in [18F]FET PET (sensitivity and specificity) and [18F]FDOPA PET (sensitivity and specificity). In order to find out the source of the heterogeneity and improve the reliability of our research results, we have adopted several strategies such as meta-regression and sensitivity analysis. For [18F] FET PET, we were unable to identify the cause of sensitivity heterogeneity through sensitivity analysis and meta-regression. One possible source of heterogeneity is the significant difference in research duration between included studies. Some studies encompassed longer follow-up periods, while others had relatively shorter intervals. This temporal variability can introduce heterogeneity in the assessment of glioma recurrence due to changes in disease progression and treatment response over time. Another contributing factor is the observation that many studies did not involve consecutive recruitment of patients. This non-consecutive recruitment approach can introduce selection bias, as patients with differing disease characteristics or treatment histories may be included, affecting the overall diagnostic accuracy. Meta-regression analysis showed that reference standard was the possible cause of specificity heterogeneity. Sensitivity analysis by excluding data from Kebir et al. (25) and Maurer et al. (24) demonstrated a combined specificity of 0.83 and 0.83 with low heterogeneity (I² = 31%, I² = 47%). This variance could be attributed to the distinct impact of various chemotherapy regimens on the frequency and characteristics of glioma pseudoprogression. For [18F]FDOPA PET, sensitivity analysis by excluding data from Rozenblum et al. (33) showed a combined specificity of 0.96, with an satisfactory heterogeneity (I² = 0%), sensitivity analysis by excluding data from Rozenblum et al. (33) yielded a combined sensitivity of 0.98, with low heterogeneity (I² = 33%), which could be explained by different cut-off thresholds. However, difference in imaging protocols, such as radiotracer dosage, imaging timing, and scanner technology, can also contribute to heterogeneity.

When assessing the advantages and disadvantages of [18F]FET PET and [18F]FDOPA PET for glioma recurrence diagnosis, it is essential to consider not only diagnostic accuracy but also practical aspects such as cost and accessibility. [18F]FDOPA PET exhibits commendable sensitivity in detecting glioma recurrence, making it a valuable tool for identifying subtle disease progression. Its ability to probe various aspects of amino acid metabolism allows [18F]FDOPA PET to effectively differentiate between active tumor tissue and treatment-related changes, enhancing diagnostic accuracy (45). But [18F]FDOPA PET can be cost-prohibitive for some healthcare systems and may be less accessible in certain regions, limiting its widespread use. While [18F]FDOPA PET has shown promise, it is relatively newer compared to [18F]FET PET, which has a longer history in clinical practice (46, 47). [18F]FET PET, while effective, may exhibit slightly lower sensitivity compared to [18F]FDOPA PET in specific cases. Because the dependence of [18F] FET on amino acid transporters may be affected by the integrity of the blood-brain barrier, in some cases affecting its accuracy (41). However, [18F] FET PET is more widespread and cheaper than [18F] FDOPA PET, making it a practical choice for many clinical environments (46). The choice between these two imaging agents should be guided by careful consideration of patient-specific factors, clinical context, cost, and accessibility. Further larger studies that focus on cost-effective comparison were needed.

[18F] FDOPA PET and [18F] FET PET are specialized forms of positron emission tomography that utilize specific radiotracers to target and visualize brain tumors. MRI, on the other hand, uses magnetic fields and radio waves to create detailed images of the brain (48).The effectiveness of [18F] FDOPA PET and [18F] FET PET lies in their ability to detect changes at a molecular level, often before these changes are visible on MRI. One study by Xiaoxue T et al. conducted a Bayesian network meta-analysis to evaluate the diagnostic accuracy of six different imaging modalities, including [18F] FDOPA PET and [18F] FET PET, for differentiating glioma recurrence from post-radiotherapy changes. The study revealed that [18F] FDOPA PET has the highest sensitivity (0.84) among the evaluated modalities, indicating its effectiveness in correctly identifying true positive cases of recurrent glioma. For [18F] FET PET, the sensitivity is 0.73, which is also relatively high, though slightly lower than [18F] FDOPA PET. MRI had the highest specificity (0.81), demonstrating its superior accuracy in correctly identifying non-recurrent cases (12). This suggests that in clinical practice, combining these imaging techniques could offer a more balanced and comprehensive diagnostic approach, utilizing the high sensitivity of PET tracers and the high specificity of MRI.

It is imperative to acknowledge the limitations of this systematic review and meta-analysis. First, only five studies provided adequate data to assess the diagnostic performance of [18F]FDOPA PET in glioma recurrence detection, resulting in a limited sample size (33–37). Second, the heterogeneity observed in our study remains a challenge that impacts the generalizability of our findings. While our sensitivity analysis and meta-regression provided valuable insights, some degree of unexplained heterogeneity still exist. Third, the diagnostic performance of [18F]FET PET and [18F]FDOPA PET may be influenced by various factors not accounted for in our analysis, such as the specific tracer dosage, timing of imaging, and variations in scanner technology. Standardization of these aspects in future research would contribute to a more comprehensive understanding of these imaging modalities.

## 5 Conclusion

In light of the findings mentioned earlier, it seems that [18F]FDOPA PET demonstrates superior sensitivity and similar specificity to [18F] FET PET. Nevertheless, it’s crucial to emphasize that [18F]FDOPA PET results were obtained from studies with limited sample sizes. Further larger prospective studies, especially head-to-head comparisons, are need in this issue.

## Data availability statement

The original contributions presented in the study are included in the article/**Supplementary Material**. Further inquiries can be directed to the corresponding author.

## Author contributions

PY: Data curation, Formal Analysis, Methodology, Software, Writing – original draft. YW: Data curation, Software, Writing – original draft. FS: Formal Analysis, Methodology, Writing – original draft. YC: Conceptualization, Supervision, Validation, Visualization, Writing – review & editing.
