# Supplementary material for: Comparing [18F]FET PET and [18F]FDOPA PET for glioma recurrence diagnosis: a systematic review and meta-analysis
Source: Front Oncol. 2024 Jan 10;13:1346951. doi: 10.3389/fonc.2023.1346951 (PMC10805829; doi:10.3389/fonc.2023.1346951)

Supplementary Fig. 1

Funnel plot evaluating publication bias in [18F]FET PET sensitivity for glioma recurrence diagnosis.


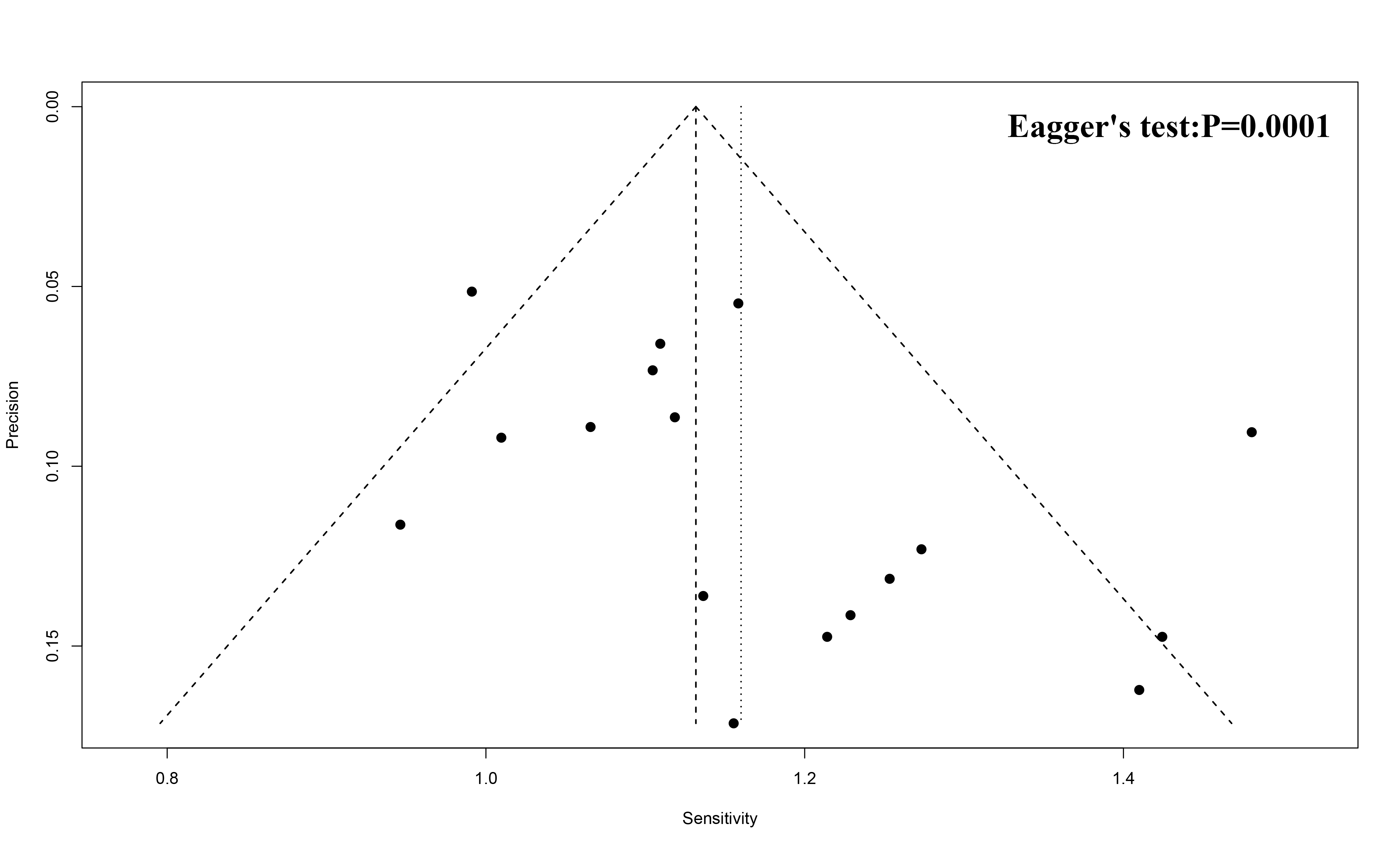


Supplementary Fig. 2

Funnel plot evaluating publication bias in [18F]FET PET specificity for glioma recurrence diagnosis.


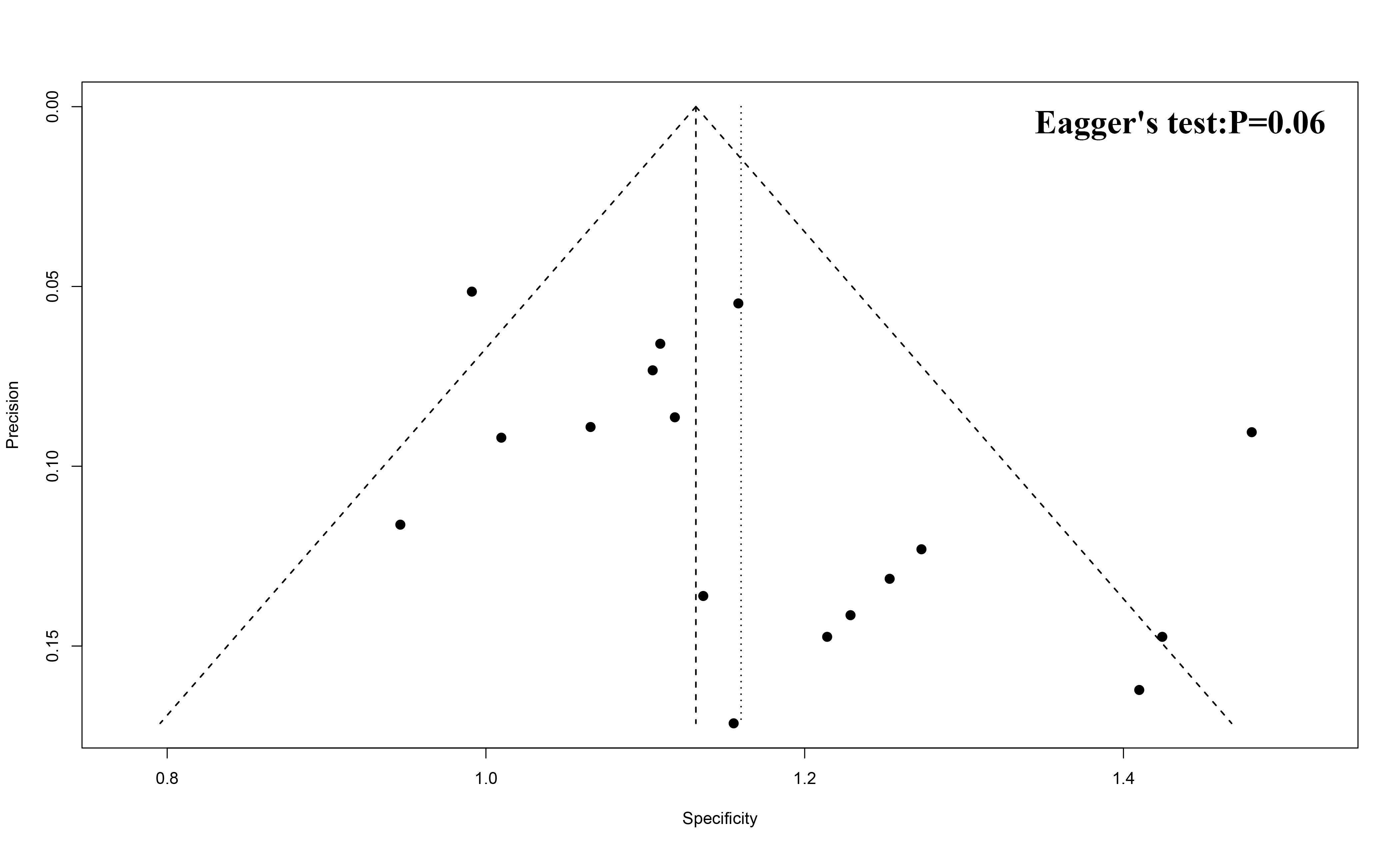


Supplementary Fig. 3

Funnel plot evaluating publication bias in [18F]FDOPA PET sensitivity for glioma recurrence diagnosis.


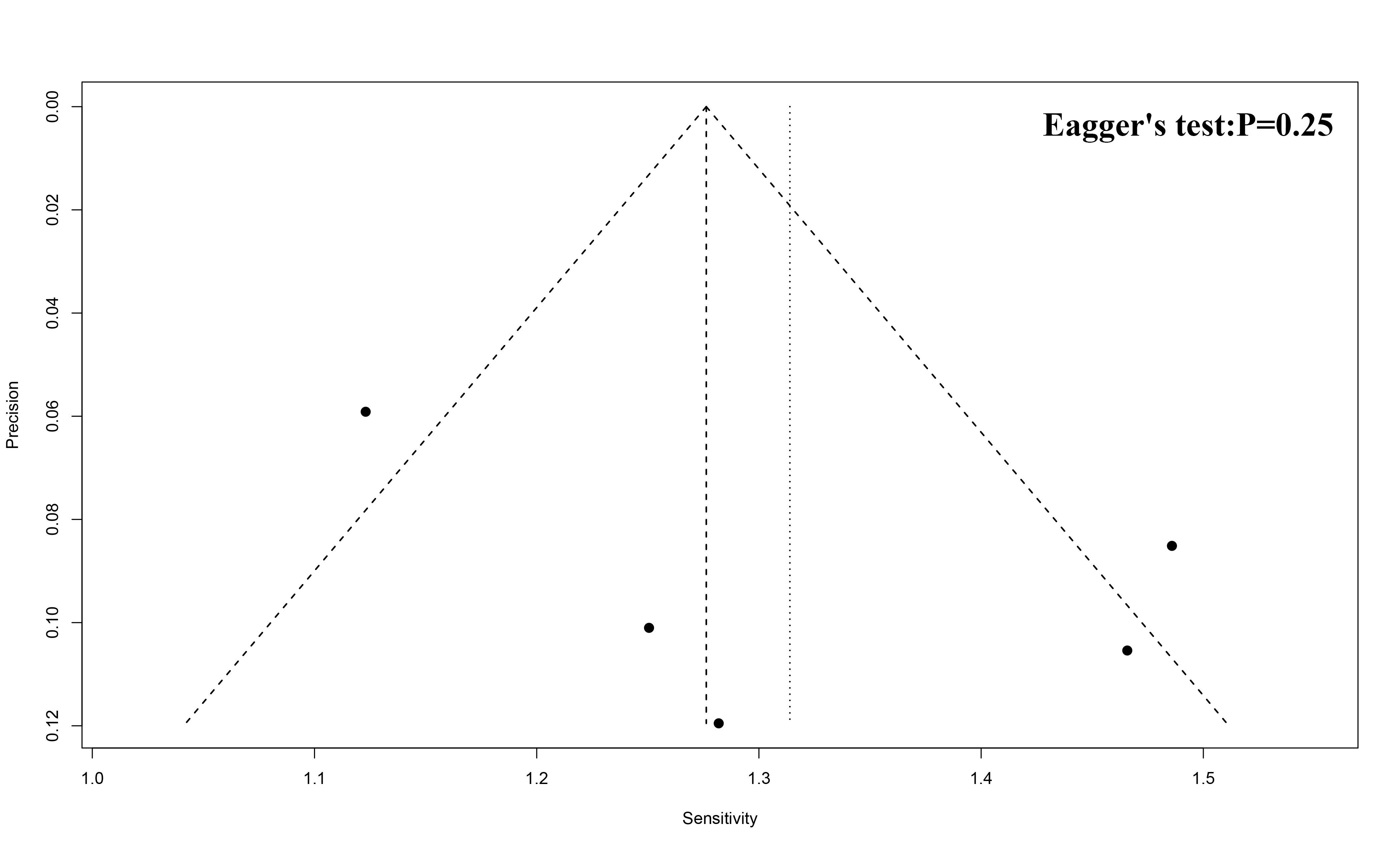


Supplementary Fig. 4

Funnel plot evaluating publication bias in [18F]FDOPA PET specificity for glioma recurrence diagnosis.


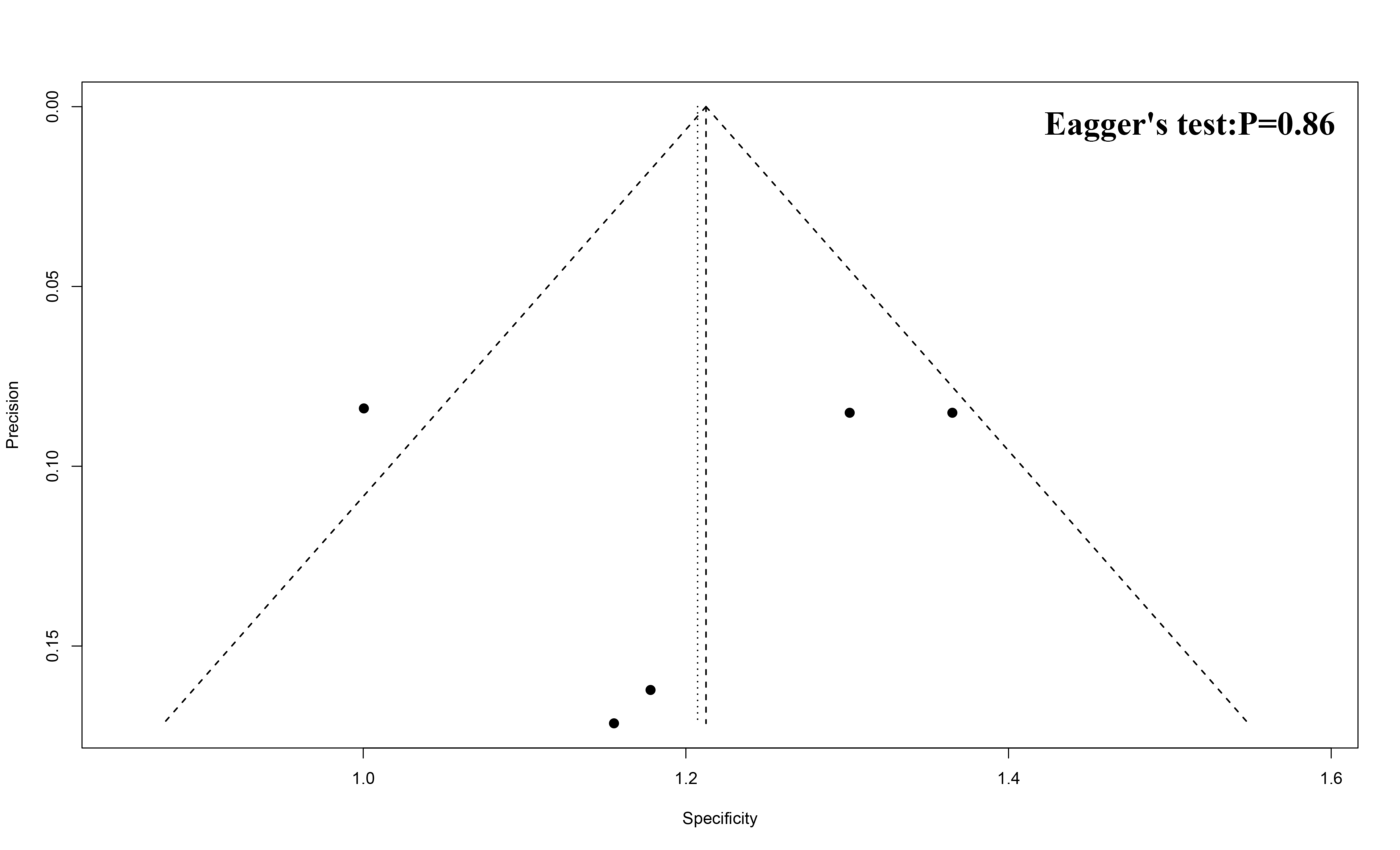

Supplement: Supplementary file 1 [file DataSheet_1.docx]
